# Triplet Therapy in Metastatic Castrate Sensitive Prostate Cancer (mCSPC)—A Potential New Standard of Care

**DOI:** 10.3390/curroncol30040332

**Published:** 2023-04-20

**Authors:** Abhenil Mittal, Srikala S. Sridhar, Michael Ong, Di Maria Jiang

**Affiliations:** 1 Division of Medical Oncology and Hematology, Department of Medicine, Princess Margaret Cancer Center, University of Toronto, Toronto, ON M5G 2C1, Canada; abhenil.mittal@uhn.ca (A.M.);; 2 Division of Medical Oncology, The Ottawa Hospital Cancer Centre, University of Ottawa, Ottawa, ON K1H 8L6, Canada

**Keywords:** metastatic castrate sensitive prostate cancer, triplet therapy, androgen receptor pathway inhibitors, docetaxel, abiraterone, darolutamide

## Abstract

The treatment paradigm for metastatic castrate-sensitive prostate cancer (mCSPC) has evolved rapidly in the past decade with the approval of several life-prolonging therapies including docetaxel chemotherapy and multiple androgen receptor pathway inhibitors (ARPI) in combination with androgen deprivation therapy (ADT). Recently reported phase-three trials have demonstrated a survival benefit of upfront triplet therapy with ADT, docetaxel plus either abiraterone acetate or darolutamide when compared to ADT plus docetaxel alone. However, multiple questions including the incremental benefit of docetaxel to a combination of ADT and ARPI, the timing of ARPI, optimal patient selection for triplet therapy and clinical and genomic biomarkers still remain to be answered. Moreover, real-world data suggest suboptimal treatment intensification with many patients treated with ADT alone highlighting challenges in implementation. In this article, we review the phase-three data associated with triplet therapy in mCSPC. We also discuss the knowledge gaps that exist despite the completion of these studies and how ongoing studies are likely to change the paradigm in the near future. Finally, we provide a simple algorithm based on current data that clinicians can use in daily practice to select patients for appropriate treatment strategies.

## 1. Introduction

Worldwide, prostate cancer is the second most common cancer among men, with more than 1.4 million cases reported in 2020 and >375,000 deaths [1]. Advanced prostate cancer is a continuum of multiple disease states, which are differentiated by their sensitivity to androgen deprivation (metastatic castrate-sensitive (mCSPC) vs. castrate-resistant prostate cancer (mCRPC)). Although a minority (~5%) of patients with prostate cancer present with synchronous (or de novo) mCSPC, this population accounts for approximately 50% of prostate-cancer-related mortality and therefore requires consideration for treatment intensification [2,3].

In mCSPC, both the timing (synchronous vs. metachronous) and disease burden of metastases (high or low volume) appear important prognostically. As per the CHAARTED criteria, high-volume disease is defined by four or more bone metastases with at least one beyond the pelvis or vertebra, or the presence of any visceral metastases [4]. Less extensive disease is otherwise classified as low-volume disease. Patients with low-volume metachronous (or recurrent) mCSPC following primary definitive therapy have the most favorable prognosis (5-year overall survival (OS) 70–75%), while high-volume synchronous mCSPC is associated with the worst prognosis (5-year OS 20–30%) [5]. These outcomes have improved over the past decade with the treatment intensification of the standard of care (SOC), using androgen deprivation therapy (ADT) plus docetaxel or an androgen receptor pathway inhibitor (ARPI) such as abiraterone acetate and prednisone (AAP), enzalutamide, or apalutamide [6]. Recently, two randomized trials have reported an OS benefit of further treatment intensification with triplet therapy (ADT plus concurrent docetaxel and an ARPI) [7,8], over ADT plus concurrent docetaxel. However, optimal patient selection for triplet therapy and treatment sequencing remains unknown. In this review, we will summarize the rationale, supporting data, and recent advances in the use of triplet therapy. We will also discuss our current treatment algorithm for patients with mCSPC.

## 2. Evolution of Systemic Therapy of mCSPC Prior to Triplet Therapy

As summarized in Table 1, the management of mCSPC has undergone a paradigm shift in the past decade. Several life-prolonging therapies that have been previously used in the mCRPC setting are now showing prolonged OS benefits when used in the earlier mCSPC setting [9]. In mCSPC, docetaxel was the first systemic therapy to show an improvement in OS when added to ADT. Although the GETUG AFU 15 study was negative for OS likely due to a greater number of low-volume patients accrued, benefits were seen in the CHAARTED study and in arm C of the multi-platform STAMPEDE trial [4,10,11,12,13]. The magnitude of survival benefit was consistent across both studies (16–17 months). In the CHAARTED study, the benefit of docetaxel was greater in patients with high-volume disease compared to low volume (HR 0.63 (0.50–0.79) in high volume and HR 1.04 (0.70–1.55) in low volume). Somewhat in contrast to CHAARTED, the STAMPEDE trial showed that disease volume did not impact the OS benefit seen when docetaxel was added to ADT. This could be due to the fact that STAMPEDE included more patients with low-volume mCSPC than CHAARTED (44% vs. 35%) [14]. Several meta-analyses including STOPCAP have shown the benefit of docetaxel was greatest in synchronous and high-volume disease [4,5,11]. As a result, ADT plus docetaxel became a SOC for high-volume mCSPC, and received a category 1 recommendation in the NCCN guidelines.

Shortly after docetaxel intensification became established, multiple independent trials evaluated ADT plus ARPIs compared to ADT alone in mCSPC (Table 1). AAP, an inhibitor of the enzyme CYP17 in the adrenal androgen synthesis pathway, was the first ARPI to receive regulatory approval in mCSPC. Both LATITUTE and STAMPEDE arm G evaluated the efficacy of AAP plus ADT vs. ADT alone and showed significant improvements in OS (HR for OS 0.66 for LATITUDE and 0.63 for STAMPEDE) [15,16]. However, these trials enrolled different populations. The LATITUDE study was restricted to patients with high-risk synchronous mCSPC (defined as two out of three of a Gleason score ≥ 8, ≥three bone lesions, and the presence of visceral metastases). On the other hand, the STAMPEDE study enrolled a broader range of patients, including 48% with nonmetastatic disease according to standard conventional imaging. In this study, post hoc analyses confirmed the efficacy of AAP across disease volumes. Subsequent trials have confirmed the benefit of apalutamide (TITAN) [18] and enzalutamide (ARCHES and ENZAMET) [19,21,22] with very similar hazard ratios for survival as AAP, and have led to regulatory approvals for these drugs across the disease spectrum of mCSPC.

An indirect comparison of AAP and docetaxel based on the results from the multi-arm STAMPEDE trial and other studies showed better failure-free survival (FFS) and progression-free survival (PFS) with AAP, but similar OS compared to docetaxel [23,24,25]. Given the absence of direct comparative data, patient selection for chemotherapy vs. ARPI remains primarily empirical. Similarly, treatment selection of any ARPI is based predominantly on the side-effect profile, patient comorbidities, and drug access in a particular healthcare setting. For instance, AAP may be less ideal in patients with uncontrolled diabetes, hypertension, or active cardiac comorbidities. On the other hand, apalutamide may be less preferred in patients with pre-existing dermatological or thyroid disorders given its propensity to cause skin rash and thyroid issues. Enzalutamide should be avoided in patients with a previous history of seizures and can cause neurological side effects including fatigue and a higher risk of falls. Overall, ADT + ARPI is the most commonly used regimen for mCSPC, given its tolerability compared to chemotherapy [26,27,28].

## 3. Triplet Therapy in mCSPC—Rationale and Summary of Clinical Data

As discussed above, numerous trials have demonstrated the significant OS benefit of moving systemic therapy earlier, from the mCRPC to the mCSPC setting. This is likely due to several reasons. mCSPC likely has more favorable disease biology, less acquired treatment resistance, and therefore demonstrates more durable treatment responses [9]. For example, long-term follow-up of the CHAARTED trial showed a sustained OS benefit with docetaxel (particularly for those with high-volume disease with 8-year OS of 28.5 vs. 15.4%) and a visible tail on the curve suggesting a long-term response in a subset of patients [29]. The use of docetaxel in mCSPC is likely more efficacious at targeting AR-independent cancer cells early, compared to its use in mCRPC, where these cancer cells may have had opportunities to develop resistance. Treatment in the mCSPC setting is also often better tolerated, given there are fewer cumulative toxicities from prior systemic therapy, as well as fewer symptoms and a lower disease burden prior to disease progression. Real-world studies have shown that, upon disease progression, only approximately 50% of patients receive second-line therapies, many of which may become too frail to receive docetaxel in the mCRPC setting. Therefore, earlier treatment intensification may also lead to more patients receiving more life-prolonging systemic therapies [30].

Despite treatment intensification beyond ADT, there remains a subgroup of patients, particularly those with poor PSA response, with suboptimal outcomes. The PSA response has been consistently shown to correlate with outcomes across trials [31]. In a post hoc analysis of the LATITUDE study, a greater PSA response (to <50% from baseline) was associated with a lower risk of progression (rPFS HR 0.26 (0.16–0.4) and death (OS HR 0.44, 0.27–0.70 [32]. The study also showed that the depth of the PSA response correlated with outcomes. Patients achieving PSA < 0.1 ng/ml had more favourable outcomes than those with >0.1 ng/ml. The rapidity of PSA response was also important with patients who achieved a PSA of <0.1 ng/ml at 6 months having better outcomes than those achieving the same response at 12 months. A longer time to PSA nadir (TPN) was associated with better rPFS and OS. The prognostic relevance of PSA nadir <0.2 was also validated by Sayegh et al. in a real-world setting [33]. Therefore, combining all three active agents (ADT, ARPI and docetaxel) in ‘triplet therapy’ could theoretically benefit these patients by addressing early disease resistance through non-overlapping mechanisms of action, and as a result, achieving a deeper PSA response and delaying disease progression.

Three major phase III trials have evaluated the role of upfront triplet therapy in mCSPC, two of which compared it to ADT plus docetaxel. ENZAMET was a phase III open-label, randomized, controlled trial testing the addition of enzalutamide to ADT for mCSPC (Table 2) [22]. After the CHAARTED results were released, the protocol was amended to allow docetaxel use at the investigator’s discretion (based on chemo-fitness and clinical appropriateness). Patients could receive two cycles of docetaxel before the commencement of enzalutamide, and most of the chemotherapy was delivered concurrently with the ARPI. Overall, 45% received concurrent docetaxel (61% of patients with high-volume disease and 27% of those with low-volume disease). The use of G-CSF was based on investigator discretion. The primary outcome of ENZAMET was OS and was stratified by docetaxel use. The most recent results after a median follow-up of 68 months (476 deaths) showed ongoing OS improvements in the overall cohort (5-year OS 67% vs. 57%, HR 0.70, *p* < 0.001) [21,34]. The benefit of enzalutamide was less pronounced in patients who received docetaxel (HR 0.82 (0.63–1.06); however, the use of docetaxel did not result in a significant interaction with the overall benefit of enzalutamide across the disease spectrum. The subgroup of patients with synchronous disease did not benefit from triplet therapy compared to ADT plus docetaxel (HR of 0.79 (0.57–1.10) for high volume and 0.57 (0.29–1.12) for low volume). For synchronous high-volume patients, there was a small early separation of curves between triplet and doublet therapy within the first 24 months, after which the curves came together, suggesting select patients may benefit from triplet therapy in this setting. However, it is important to note that ENZAMET was not powered for this type of exploratory analysis, and confounding by indication may exist (patients selected for docetaxel could have had inherently worse disease outcomes than patients who were not selected for chemotherapy). Moreover, there were higher treatment discontinuations in the enzalutamide arm (33% vs. 14%), and only 65% of patients completed the planned six cycles of docetaxel, further complicating the interpretation of these findings. The toxicity of the triplet combination of docetaxel and enzalutamide will likely be more pronounced in real-world patients who are less fit compared to the trial patient population and therefore will require careful patient selection.

PEACE-1 was a phase III open-label, randomized controlled trial with a 2 × 2 factorial design, which randomized patients with synchronous mCSPC to receive SOC, SOC + AAP, SOC + prostate radiotherapy, or SOC + AAP + prostate radiotherapy. The arms evaluating prostate radiotherapy were pooled with corresponding systemic therapy arms to analyze the benefit of AAP + SOC vs. SOC. The trial began accruing in 2013 when ADT alone was the SOC [7] and underwent several protocol amendments to account for evolving SOC during trial enrollment. With the publication of CHAARTED and STAMPEDE data [10,11], concurrent docetaxel with ADT was allowed at the investigator’s discretion and later made mandatory in the SOC arm. Primary G-CSF prophylaxis was also made mandatory after an amendment in 2018. The study was powered for co-primary end points for radiographic PFS (rPFS) and OS. Multiple secondary end points including CRPC-free survival, time to pain progression, time to chemotherapy for CRPC, prostate-cancer-specific survival, and quality of life, were also included. Analyses were stratified by several variables including docetaxel use. The trial enrolled a relatively high-risk population, which included 63% high volume and 11% with visceral metastases. Overall, 60% of patients received concurrent docetaxel with ADT (the first cycle 6 weeks to 3 months after initiation of ADT) and AAP (started within 6 weeks of ADT). In the docetaxel subgroup, the addition of AAP (triplet therapy) had a significant rPFS benefit (4.5 years vs. 2 years, HR 0.50 (0.40–0.62), *p* < 0.0001), which were similar irrespective of disease volume. The addition of AAP also led to a significant OS improvement (median OS not reached vs. 4.5 years, HR 0.75, 0.59–0.95, *p* = 0.017), especially in patients with high-volume disease (median OS 5.1 vs. 3.5 years, HR 0.72 (0.55–0.95), *p* = 0.019). The data for patients with low-volume disease remains immature given that there have been very few events to date. There were also improvements in secondary end points such as time to CRPC and prostate-cancer-specific survival. No clear increase in grade 3–4 adverse events was observed with the use of triplet therapy, except for a slightly higher incidence of liver function test abnormalities and hypertension. The neutropenia (10% vs. 9%) and febrile neutropenia (5% vs. 5%) rates were similar. Overall, patients received a median of all six cycles, and the triplet regimen was very well tolerated.

ARASENS evaluated the addition of darolutamide (a second-generation non-steroidal AR antagonist) to docetaxel and ADT in patients with mCSPC [8]. This was a global phase III randomized placebo-controlled trial that enrolled patients between 2016 and 2018 after docetaxel became a SOC. The use of growth factor support with docetaxel was not specified in the protocol. The primary end point was OS. rPFS was neither a primary nor secondary endpoint as imaging was not mandated every 12 weeks. Instead, patients were evaluated every 12 weeks for secondary endpoints including castration resistance, skeletal events, opioid use, and pain progression. The study population included 86% with synchronous metastatic disease. The proportion of patients with visceral disease was slightly higher in ARASENS compared to PEACE-1 (17.5% vs. 12%). Approximately 77% of patients had high-volume disease (the majority with bone metastasis with 23% high volume having visceral metastasis) [35]. Most patients completed six cycles of docetaxel (87.6% in the darolutamide group and 85.5% in the placebo group). The trial demonstrated a significant improvement in OS with the addition of darolutamide (median OS not reached vs. 48.9 months, HR 0.68, 0.57–0.80, *p* < 0.001), despite 75.6% of patients in the placebo arm receiving subsequent life-prolonging systemic therapy. Secondary end points including the time to development of castration resistance, time to pain progression, time to skeletal events, and time to subsequent neoplastic therapy were also significantly improved in the triple therapy arm. Data by disease volume were recently presented at the American Society of Clinical Oncology Genitourinary (ASCO GU) symposium 2023 and subsequently published and showed a statistically significant OS benefit in patients with high-volume disease (HR 0.69, 0.57–0.82). In the low-volume group, median survival was not reached in either arm, and the HR was suggestive of a benefit but not statistically significant (HR 0.68, 0.41–1.13) (35) There were no additional safety signals identified, and no increase in serious adverse events was noted in the triple therapy arm compared to the doublet. Rates of febrile neutropenia (7%) were similar to those reported with docetaxel in the PEACE-1 study.

## 4. Putting the Evidence into Perspective

Although level I evidence supports triplet therapy in mCSPC with the addition of ARPI to a backbone of ADT + docetaxel, the most controversial question surrounds the magnitude of survival benefit gained by adding docetaxel to a backbone of ADT + ARPI [26,27,28]. Until a trial is completed on a backbone of ADT + ARPI and randomizing patients to docetaxel, clinicians will need to decide whether they offer triplet therapy instead of ADT + ARPI without level I evidence of benefit, and to which patients. ADT + ARPI reaches a wide population of patients since subgroups of low-volume, high-volume, high-risk, low-risk, synchronous, and metachronous metastases categories will benefit from treatment, and most patients can tolerate it without major issues. In contrast, the addition of docetaxel will need more careful considerations including disease factors (such as metachronous vs. synchronous presentation, the volume of disease, risk category, the presence of visceral disease and histological and molecular risk factors) and patient factors (chronological age, performance status, organ function, comorbidities such as diabetes neuropathy, and risk factors for infection). The ENZAMET data suggest many patients with synchronous high-volume disease do well with ADT-ARPI alone, and the addition of concurrent docetaxel may not further improve outcomes [21].

Multiple systematic reviews and network meta-analyses have attempted to indirectly compare ADT + ARPI + docetaxel vs. ADT + ARPI based on the randomized trials reviewed and also including other RCTs studying ADT + ARPI vs. ADT alone [36,37,38,39,40,41]. These meta-analyses have shown mixed results regarding the benefit of docetaxel [36,37,38,40,41] in the context of inherent biases in the absence of individual patient data for definitive comparisons. Although conducting a formal clinical trial to define the benefits of triplet therapy is ideal, there may be challenges to trial accrual given that ADT + ARPI is a highly effective backbone of treatment; many men with prostate cancer may be reluctant to be randomized to receive chemotherapy; and many clinicians may inherently favour either a doublet or triplet approach depending on patient characteristics and tumour risk factors. The available data support the use of triplet therapy in high-volume rather than low-volume mCSPC [7,35], but this definition imperfectly captures the presence of any AR-independent clones, which would benefit from adding docetaxel. Another prognostic factor for docetaxel triplet selection may include synchronous rather than metachronous disease. However, it is currently unknown whether patients with metachronous high-volume disease can also benefit due to the small number of patients included in studies [29,35]. Notably, patients with visceral metastases continue to have poor outcomes with ADT and ARPI across multiple studies: TITAN (HR 0.71, 0.43–1.18) [18], ARCHES (HR 1.16, 0.67–2.00) [20], and ENZAMET studies (HR 1.05, 0.54–2.02) [22]. These patients may have greater benefits from triplet therapy. A recent exploratory analysis of the PEACE-1 study presented at the European Society of Medical Oncology (ESMO) 2022 showed that triplet therapy significantly improved the rPFS of patients with liver metastases compared to ADT plus docetaxel (HR 0.3, *p* = 0.02), with a trend for improved survival (median OS 3.2 vs. 2.5 years, HR 0.5, *p* = 0.12) [42,43]. In a further exploratory subgroup analysis of high-volume patients from ARASENS, there was a trend towards improvement in survival with triplet therapy in patients with visceral metastasis (median OS 49 months vs. 42 months, HR 0.79, 0.55–1.14) with overall inferior outcomes than in those with bone-only disease. However, given the small sample sizes, further prospective data are required. Interestingly, not all visceral metastases are the same prognostically. Although often grouped in the same category, lung metastases may be associated with better outcomes compared to liver metastases [44]. This suggests further treatment stratification is needed in future clinical trial design. Active symptoms such as pain at presentation also have been shown to be an independent prognostic factor for survival; however, its relevance in clinical decision making remains to be determined [24].

It also remains unclear whether all three drugs need to be given concurrently or if ARPI can be started before or after docetaxel. Unfortunately, very few patients have been treated with a sequential triplet strategy in these trials, with the use of ARPI following docetaxel (e.g., in the TITAN trial, only 10% of patients received sequential triplet therapy) and the benefit of this sequential approach vs. concurrent treatment remains unknown. A recent meta-analysis suggested that the benefit of triplet therapy was restricted to the concurrent administration of all three drugs; however, a small number of patients in individual trials limit definite conclusions [39]. In a clinical setting, drug access to ARPI may limit the feasibility of concurrent administration of triplet therapy.

Currently, validated predictive molecular biomarkers remain lacking in clinical practice. A recent analysis by Swami et al. showed that mutations in *SPOP* were associated with the benefit of the addition of ARPI rather than docetaxel, which represents a potential biomarker for treatment selection of doublet therapy if validated [45]. Other studies have shown that patients with high-volume disease tend to have mutations in DNA damage repair or the androgen receptor, rendering them less sensitive to ARPI and more likely to benefit from the addition of docetaxel [46,47,48,49]. Transcriptomic profiling suggests that luminal but not basal subtypes seem to benefit from upfront chemotherapy [50]; however, these markers need to be validated in larger studies and demonstrate feasibility in real-world settings before they can be incorporated into routine clinical practice. Biomarker analysis from PEACE-1 and ARASENS trials are eagerly awaited to further inform patient selection for triplet therapy.

## 5. Clinical Practise Points

**Figure 1:**
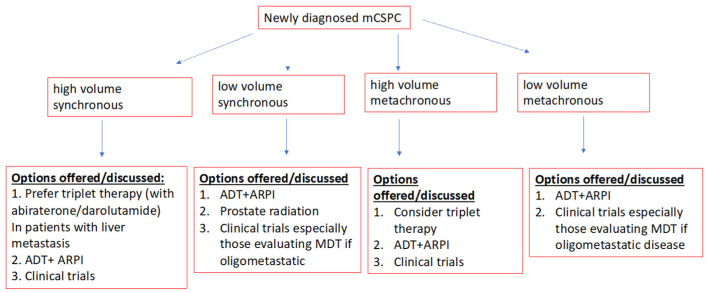
Proposed patient selection algorithm for patients with mCSPC. CSPC—castrate sensitive prostate cancer, ADT—androgen deprivation therapy, ARPI—androgen receptor axis targeted therapy, MDT—metastasis directed therapy.

The goal of treatment intensification in the mCSPC setting includes delaying disease progression to mCRPC, improving overall survival, and maintaining the quality of life. Figure 1 represents a practical guide on how we select treatment options for patients with mCSPC. Clinical trials are offered whenever possible. For patients who are planned for docetaxel, triplet therapy with the addition of either AAP or darolutamide should be used given improved overall survival, and ADT + docetaxel can no longer be considered a standard of care. Triplet therapy should be considered in fit men with high-volume synchronous mCSPC, especially in those with visceral disease. Although the data on G-CSF utilization rates in triplet trials are not yet available, primary prophylaxis with G-CSF or peg-GCSF is reasonable given our own institutional experience in preventing neutropenia and hospital admissions [51]. ADT plus ARPI is a reasonable option for patients who are not chemo fit, elderly, chemo averse, or have bone-only disease.

For patients with low-volume disease, we do not routinely offer triplet therapy. These patients are primarily managed with ADT + ARPI (either AAP, enzalutamide or apalutamide based on the toxicity profile, patient comorbidities and access) and prostate primary radiation. In those with metachronous disease, participation in clinical trials evaluating metastases-directed therapy should be encouraged if available. Very few patients with mCSPC should be treated with ADT alone given most are able to tolerate ARPI. 

Currently, the presence of metastases and definitions of disease volume for treatment intensification for mCSPC are based on conventional imaging (CT and bone scan). PSMA PET imaging is not approved in this setting as none of the landmark trials included this in their protocols. PSMA PET imaging is anticipated to detect more metastases than conventional imaging [52,53]; however, there are no data to suggest their use for staging mCSPC can improve outcomes. There is also a potential risk for classifying low-volume to high-volume disease, which can lead to both over-treatment (adding docetaxel) and under-treatment (omitting prostate primary radiation, which is associated with an overall survival benefit). Therefore, this approach cannot be recommended at this time without further supporting prospective data. 

## 6. Future Directions

A number of trials evaluating biomarker-guided and targeted therapeutic approaches are ongoing and may further change the current treatment paradigm of mCSPC (Table 3). These concepts are being studied in the AMPLITUDE [54] and TALAPRO-3 [55] trials testing the efficacy of PARP inhibitors niraparib and talazoparib, respectively, in addition to ARPI and ADT in patients with homologous recombination repair (HRR) pathway defects. Other trials are evaluating the utility of targeting the PTEN pathway (the CAPITELLO-281 trial using Capivasertib [56]), the CDK4/6 cell cycle (the CYCLONE-3 trial using Abemaciclib) [57], and others. Studies evaluating immune checkpoint inhibitors in combination with ADT and ARPI are also ongoing [58].

Radiotherapy and radio-nucleotide therapies are also being rigorously evaluated in mCSPC (e.g., PSMAddition trial) [59]. Trials incorporating PSMA-PET imaging to detect lesions occult on conventional imaging and to intensify treatment in the castrate-sensitive setting are also ongoing [60,61]. Metastasis-directed therapy (MDT) is emerging as a promising treatment option for patients with metachronous oligometastatic prostate cancer [62,63], and its role in mCSPC in addition to ADT plus enzalutamide will be assessed by studies including the phase-three SPARKLE study [64].

**Table 3:** Select ongoing trials in mCSPC.

	Keynote 991 [58]	PSMAddition [59]	AMPLITUDE [54]	TALAPRO-3 [55]	CAPITELLO-281 [56]	CYCLONE-3 [57]	SPARKLE [64]
NCT number	NCT04191096	NCT04720157	NCT04497844	NCT04821622	NCT04493853	NCT05288166	NCT05352178
Experimental arm	Pembrolizumab plus Enzalutamide plus ADT	Lu-177 plus SOC	Niraparib plus AAP plus ADT	Talazoparib plus enzalutamide plus ADT	Capivasertib plus AAP plus ADT	Abemaciclib plus AAP plus ADT	1 = MDT plus 1 month ADT 2 = MDT plus 6 months ADT + enzalutamide
Control arm	Enzalutamide plus ADT	SOC alone	AAP plus ADT	Enzalutamide plus ADT	AAP plus ADT	AAP plus ADT	MDT alone
Design	Randomised phase III double blind	Randomised phase III with cross over allowed	Randomised phase III double blind	Randomised phase III double blind	Randomised phase III double blind	Randomised phase III double blind	Randomised phase III open label
Number of patients	1232	1126	788	550	1000	900	873
Primary end point	rPFS and OS	rPFS	rPFS	rPFS	rPFS	rPFS	Poly metastatic free survival (PMFS)
Current status	Active, not recruiting	Recruiting	recruiting	Completed recruiting	Recruiting	recruiting	Recruiting

AAP—abiraterone prednisone, SOC—standard of care, ADT—androgen deprivation therapy, rPFS—radiographic progression-free survival, OS—overall survival. Source: clinicaltrials.gov.

More data on real-world outcomes are also anticipated as initial studies suggest that real-world intensification rates in mCSPC are exceedingly poor. Given that >50% of patients with synchronous mCSPC are still treated with ADT alone in the real world [65,66,67], further research is warranted to evaluate potential barriers of treatment intensification and whether there are improvements over time in both academic and community settings.

## 7. Conclusions

The treatment landscape for mCSPC has evolved quickly. Recently, data from multiple phase III trials have supported the use of triplet therapy over ADT plus docetaxel, in patients with mCSPC and unfavourable disease biology. Patients who are chemo fit with liver metastases should be considered for triplet therapy. However, many unanswered questions remain, including the benefit of triplet therapy over ADT plus ARPI, the timing of these systemic therapy agents, and optimal treatment selection. The uptake of treatment intensification in the mCSPC setting continues to be poor in the real-world setting. Ongoing studies evaluating a molecular select approach for targeted therapeutic strategies are underway, which will hopefully translate into improved outcomes.

## Figures and Tables

**Table 1:** Summary of trials evaluating doublet therapy in mCSPC.

	CHAARTED [4,11]	STAMPEDE (DOC) [10]	LATITUDE [15]	STAMPEDE (AAP) [14,16,17]	TITAN [18]	ARCHES [19,20]
*N*	790	1776 (61% of patients with mCSPC)	1199	1917	1052	1150
Treatment arms	ADT + docetaxel ADT	SOC (ADT) SOC + docetaxel	ADT + abiraterone + prednisone ADT + placebo	SOC (ADT) SOC + abiraterone + prednisolone	ADT + apalutamide ADT + placebo	ADT + enzalutamide ADT + placebo
Disease risk	65% high volume	56% high burden	100% high risk	52% high risk (among M1)	63% high volume	63% high volume
Synchronous	73%	58%	100%	49%	81%	67%
Primary end point	OS: HR 0.72 (0.59–0.89), *p* = 0.0017	OS: HR 0.78 (0.66–0.93), *p* = 0.006 OS: HR 0.76 (0.62–0.92) for M1, *p* = NS	OS: HR 0.66 (0.51–0.76), *p* < 0.0001 rPFS: HR 0.47 (0.39–0.55), *p* < 0.001	OS: HR 0.61 for M1, *p* = 0.005	rPFS: HR 0.48 (0.39–0.60), *p* < 0.0001 OS: HR 0.65 (0.51–0.89), *p* < 0.0001	rPFS: HR 0.39 (0.30–0.50), *p* < 0.0001 OS (final analysis): HR 0.66 (0.53–0.81), *p* < 0.0001
High risk/High volume	OS: HR 0.63 (0.50–0.79), *p* < 0.001	OS: HR 0.81 (0.64–1.02), *p* = 0.064	OS HR: 0.58 (0.41–0.83)	OS: HR 0.54 (0.41–0.70), *p* < 0.05	OS HR: 0.68 (0.50–0.92) rPFS HR: 0.53 (0.41–0.67)	OS: HR 66 (0.52–0.83)
Low risk/Low volume	OS: HR 1.04 (0.70–1.55), *p* = 0.68	OS: HR 0.76 (0.54–1.07), *p* = 0.207	OS HR: 0.69 (0.58–0.82)	OS: HR 0.66 (0.44–0.98), *p* < 0.05	OS: HR0.67 (0.34–1.32) rPFS: HR 0.36 (0.22–0.57)	OS: HR 0.66 (0.43–1.02)
QOL	Worse for ADT + docetaxel at Month 3 (FACT-P = 116.3 vs. 118.3) but better by Month 12 (FACT-P = 119.2 vs. 116.4)		Improved for ADT + abiraterone + prednisone		No difference/ Maintained	No difference/ Maintained
Select adverse events (Gr ≥ 3)	Febrile neutropenia: 6%	Febrile neutropenia: 15%	Grade 3/4: 68% Most common: hypertension, hypokalemia	Grade 3–5: 47% Most common: endocrine, cardiovascular disorders	Grade 3–4: 42.2% Most common: hypertension, rash	Grade ≥ 3: 24.3% Most common: hypertension, fatigue

ADT—androgen deprivation therapy, SOC—standard of care, OS—overall survival, PFS—progression-free survival, CSPC—castrate sensitive prostate cancer, NA—not available, FACT-P—Functional Assessment of Cancer Therapy-Prostate.

**Table 2:** Summary of trials evaluating triplet therapy trials in mCSPC.

	ARASENS [8,35]	PEACE-1 [7]	ENZAMET [21,22]
*N*	1306	1173	1125
Treatment arms	ADT + Docetaxel + Darolutamide vs. ADT + Docetaxel	SOC vs. SOC + AAP (SOC included docetaxel in 710 patients)	ADT + enzalutamide ADT + NSAA
Disease volume	77% high volume	64.2% high volume	52% high volume
Synchronous	86.1%	100%	61%
Primary end point	OS	rPFS and OS	OS
Primary end point	OS: HR 0.68 (0.57–0.80), *p* < 0.001	rPFS HR: 0.50 (0.40–0.62), *p* < 0.0001 OS: HR 0.75 (0.59–0.95), *p* = 0.017	OS: HR 0.67 (0.52–0.86), *p* = 0.002
Key Secondary end points	Time to CRPC: HR 0.36 (0.30–0.42), *p* < 0.0001	CRPC free survival: HR 0·38, 95% CI 0·31–0·47; *p* < 0·0001 Prostate cancer specific survival: HR 0·69, 95% CI 0·53–0·90; *p* = 0·0062	PFS; HR 0.40 (0.33–0.49), *p* < 0.001
High volume	OS HR: 0.68 (0.57–0.82)	OS: HR 0.72 (0.55–0.95), *p* = 0.019	NA
Low volume	OS HR: 0.68 (0.41–1.13)	OS: HR 0.83 (0.50–1.38), *p* = 0.66	NA
QOL	Not reported yet	Not reported yet	No difference/ Maintained
Select adverse events (Gr ≥ 3)	Febrile Neutropenia rates: 7.8% vs. 7.4% Hypertension- 6.4% vs. 3.2% Increased liver enzymes- 5.4% vs. 2.8%	Febrile Neutropenia 5% with docetaxel Hypertension- 22% with AAP vs. 13% in SOC Hepatotoxicity 6% in AAP vs. 1% in SOC	Enzalutamide + docetaxel, 65% completed planned 6 cycles Among patients who received docetaxel, >grade2 neuropath 9% with docetaxel and 3% without

ADT—androgen deprivation therapy, SOC—standard of care, OS—overall survival, PFS—progression-free survival, CSPC—castrate sensitive prostate cancer, NA—not available, AAP—abiraterone acetate and prednisone.
